# Draft genome sequences of clinical *Mycobacterium canettii* strains in Canada

**DOI:** 10.1128/mra.00622-24

**Published:** 2024-09-19

**Authors:** Md Rashedul Islam, Heather Adam, Pierre-Marie Akochy, Meenu Sharma, Alisa McGurran, Hafid Soualhine

**Affiliations:** 1 National Reference Centre for Mycobacteriology, National Microbiology Laboratory, Public Health Agency of Canada, Winnipeg, Manitoba, Canada; 2 Shared Health, Winnipeg, Manitoba, Canada; 3 Department of Medical Microbiology and Infectious Diseases, Max Rady College of Medicine, University of Manitoba, Winnipeg, Manitoba, Canada; 4 Laboratoire de Santé publique du Québec, Montreal, Québec, Canada; Wellesley College Department of Biological Sciences, Wellesley, Massachusetts, USA

**Keywords:** *Mycobacterium canettii*, genome, human tuberculosis, drug susceptibility testing

## Abstract

*Mycobacterium canettii* is a rare pathogen causing tuberculosis in humans and presents a risk to public health. Here, we report the genome sequences of two *M. canettii* strains. The genomes will assist in creating sequence-based tools for *M. canettii* and serve as references for identification, surveillance, and epidemiological investigations.

## ANNOUNCEMENT

*Mycobacterium canettii*, which was first described in 1969 (1), is a smooth tubercle bacillus that causes tuberculosis in humans, and infections by this pathogen are mainly reported in the Horn of Africa (2, 3). However, many of the geographical and biological characteristics underlying *M. canettii* are largely unknown. In this study, two *M. canettii* strains 1901080 and 2000326 were isolated from the sputum and cervical lymph node samples of humans in the Canadian province of Quebec in 2019 and Manitoba in 2020, respectively. The provincial laboratories grew the cultures at 37°C in the BACTEC MGIT tube after 2 weeks of incubation in the BD BACTEC MGIT 960 System and forwarded the cultures to the National Reference Centreer for Mycobacteriology (NRCM), Winnipeg for identification. At the NRCM, the cultures were grown aerobically at 37°C in BACTEC MGIT 960 media for a week and on Middlebrook 7H10 agar plates for 2–3 weeks. Colony morphology of the cultures was smooth, and the identification was confirmed by the presence of the RD9 region (4), a silent mutation in the *pncA* gene (Ala46Ala) (5), and other *M. canettii*-specific mutations within *gyrB, LeuS,* and *mmpL6* genes (6). Phenotypic drug susceptibility testing (DST) was performed using BACTEC 960 System. Genomic DNA was extracted using the InstaGene Matrix (6) comprising 6% (wt/vol) Chelex resin for PCR-ready DNA purification (Bio-Rad#7326030; California, United States). The sequencing library was constructed using the Illumina DNA Prep Kit (Illumina, California, United States), following the manufacturer’s protocol. Paired-end sequencing was performed on an Illumina MiSeq platform (Illumina Inc.) using a 300-cycle MiSeq Reagent Kit v2 to generate 2 × 150 bp paired-end reads. Raw sequencing reads were quality-checked using a Galaxy (7) workflow “Sequencing Report v.2.3,” where FastQC (8) and SMALT (https://www.sanger.ac.uk/tool/smalt-0/) are the main tools. The reads were processed in the IRIDA (9) by Shovill v.1.0.4, which used SPAdes v.3.13.0 for *de novo* assembly (10), and QUAST for assembly assessment (11). The genomes were annotated using the National Center for Biotechnology Information (NCBI) Prokaryotic Genome Annotation Pipeline 2023–10-03.build 7061 (12), and the Proksee server (13). Mykrobe v.0.10.0 (14) and TBProfiler v.4.4.1 (15) were used for predicting the antimicrobial resistance. Default parameters were used for all software, unless otherwise mentioned.

Table 1 shows that the genomes of *M. canettii* strains 1901080 and 2000326 contain 4,258 and 4,185 predicted coding DNA sequences, respectively, with 65.5% G + C content and 45 tRNAs. While 1901080 contains two copies of the 5S, 16S, and 23S rRNA genes and a clustered regularly interspaced short palindromic repeats (CRISPRs), 2000326 has one copy of the mentioned rRNA genes and two CRISPR arrays. FastANI (v1.33) (16) whole-genome comparison of 1901080 and 2000326 with a reference *M. canettii* CIPT140010059 (17) created an average nucleotide identity of 99.64% and 98.65%, respectively. Both strains were phenotypically mono-resistant to pyrazinamide and predicted to be susceptible to isoniazid, rifampicin, and ethambutol.

**TABLE 1:** Characteristics of the genome sequences of *Mycobacterium canettii*

Strain name	No. of reads	Coverage (X)	Assembly size (bp)	N50 (bp)	No. of contigs	No. of CDs	No. of genes	No. of rRNAs	No. of tRNAs	Genome accession no.
1901080	1,475,268	45.98	4,421,382	64,417	224	4,258	4,312	6	45	JBEEEP000000000
2000326	2,726,726	83.30	4,368,268	58,431	231	4,185	4,236	3	45	JBEEEO000000000

## Data Availability

The raw reads and assembled genome sequences have been deposited in the NCBI database under BioProject PRJNA1109359. The accession numbers for raw and assembled data are SRR29477259 and SRR29477258 and JBEEEP000000000 and JBEEEO000000000 (Table 1).
